# Home but still engaged: participation in social activities among the homebound

**DOI:** 10.1007/s11136-016-1245-2

**Published:** 2016-02-17

**Authors:** Sarah L. Szanton, Laken Roberts, Bruce Leff, Janiece L. Walker, Christopher L. Seplaki, Tacara Soones, Roland J. Thorpe, Katherine A. Ornstein

**Affiliations:** Johns Hopkins University, Baltimore, MD USA; University of Rochester, Rochester, NY USA; Mount Sinai Medical Center, Chicago, IL USA

**Keywords:** Homebound older adults, Community participation, Favored activities

## Abstract

**Purpose:**

Participation in social and community activities that require leaving one’s home is important to older adults; however, many older adults have difficulty or are unable to leave their dwellings, and little is known from national samples about issues related to remaining active outside the home or the barriers faced by these older adults.

**Design and methods:**

We used the National Health and Aging Trends Study, a nationally representative study of older adults (*n* = 7197), to understand the following: (1) the importance that homebound and semi-homebound adults place on involvement in social or community activities, (2) their current level of involvement, and (3) reported barriers to participation.

**Results:**

Despite the heavy burden of functional limitations, depression, pain, and falls, homebound adults reported that activities outside the home were important to them ranging from 25.2 % (attend clubs) to 70.0 % (visit family). Similarly, semi-homebound older adults had a strong interest in such participation, including visiting friends and family (81.8 %), attending religious services (72.6 %), and going out for enjoyment (72.5 %). Many homebound adults reported health (42.9–64.1 % depending on the activity) and transportation (12.2–18.2 %) as barriers to participation. Semi-homebound adults also identified health (23.8–41.0 %) and transportation (6.5–10.2 %) as participation barriers.

**Implications:**

This information can be useful in designing community programs that will foster meaningful social and community engagement for older adults, which may improve their quality of life.

## Introduction

Participation in community and family activities is an important aspect of quality of life for older adults. The social participation literature shows that social participation is related to better functional skills [1, 2], health-related quality of life [3, 4], and even survival [3, 5–7]. The favorite activities of older adults of almost any age include physical activities and activities that require leaving the house [8]; however, many older adults have difficulty or are unable to leave their dwellings.

The homebound are increasingly recognized as a population of special needs [9]. In the context of understanding the vital importance of community participation, it is important to study such participation in older homebound people. They are on a trajectory of decline in which inability to participate accelerates. The participation itself provides activation and motivation to prolong participation.

There has been prior quantitative and qualitative work on community barriers to participation by homebound older adults in small, geographically restricted samples. Sanders et al. [10] found that among an all-female sample of homebound older adults in one housing complex in Canada (*n* = 33), access to and cost of transportation, knowledge of available programming, and ability to access the programs offered limited activity participation. Bendixen et al. [11] in a sample in western New York State (*n* = 616) found that older adults were limited by transportation, poor health, lack of companionship, and accessibility. In a qualitative study of disabled older adults, Turcotte et al. [12] (*N* = 33) found that lack of social activities was the largest source of unmet need. However, little is known from nationally representative samples about what kind of social and community participation is important to this population of older adults and what issues they face in remaining active outside their homes. As locality, climate, supports, and building design may differ across regions of the USA, examining this issue in a nationally representative sample will provide a much-needed overview on the state of the homebound in the USA.

*We used the NHATS sample to examine issues for homebound in the USA.* In examining the association between homeboundedness and community participation, Verbrugge and Jette’s [13] disablement theory posits that both intrinsic and extrinsic factors contribute to the development of impairment from pathology. According to their theory, intrinsic characteristics such as pain as well as extrinsic factors such as community services are separate domains with different intervention targets on the pathway to disability. *It is therefore important to* examine both intrinsic and extrinsic barriers to participation among the homebound.

*The conceptual framework for disability used in the NHATS study is Freedman’s framework* [14]. *This conceptual framework for disability* advances the work of the Nagi model [15] and the World Health Organization International Classification of Functioning model [16] to support investigations of the participation of older adults in social and community activities. This *advanced* framework, which undergirds the National Health and Aging Trends Study (NHATS), serves four key functions: (1) allows for the study and consequences of participation; (2) explicitly includes testable links between the physical and social environment, participation, and disability; (3) supports research focusing on maximization of function in any stage of the disablement process; and (4) distinguishes between the capacity to perform and the actual performance, which allows study of assistive devices and environmental changes.

Thus, we apply the NHATS conceptual framework to test hypotheses about the importance of community and social participation of homebound older adults and the barriers to such participation. We hypothesized that the majority of homebound older adults would report that community participation was important but that both intrinsic (individual) and extrinsic (environmental) factors present barriers to participation among older adults. We currently know very little through nationally representative samples about outside activities older adults wish to engage in and what issues they face in remaining active outside their homes. Understanding this information can be useful to designing appropriate home and community-based social support programs that facilitate meaningful social and community engagement for a full range of older adults rather than just for physically robust ones.

## Methods

### Study sample

We used data from the NHATS survey, a longitudinal nationally representative survey of Medicare beneficiaries 65 years of age and older with information on late-life functioning, economic and social well-being, and quality of life factors of aging. Beginning in 2011, annual in-person interviews were conducted with respondents and/or proxies selected from the Medicare enrollment database using a stratified three-stage sample design, with oversampling of older age groups and Black, non-Hispanic individuals. The baseline response rate was 71 %, yielding a total sample of 8245 respondents [17]. For our analyses, we used data from Round 1 of the NHATS survey. Our analytic sample included only community-dwelling older adults (*n* = 7197), which excluded persons living in facilities—either assisted living or nursing homes—due to our focus on understanding the lives of those aging at home. Participants gave informed consent, and ethical approval for the study was given by the Institutional Review Board of the Johns Hopkins Medical Institutions.

### Measures

#### Main exposure: homebound status

We analyzed homebound status using three groups—the homebound, semi-homebound, and not homebound—using the question, “Within the last month, how often did you go outside?” Individuals were classified as homebound if they answered “Never” or “Rarely” (once a week or less). Respondents were considered semi-homebound if they went out two or more days a week, but never by themselves, needed help, or had difficulty leaving the home. The non-homebound went out two or more days per week, never needed help, and did so without difficulty. These delineations reflect the established measures of homebound status developed by Ornstein et al. [9]. This variable is considered ordinal.

#### Covariates: demographic measures

Each respondent’s birthdate was confirmed with the participant and used to calculate age at interview. Race was self-reported, and answers were reduced to four categories: non-Hispanic White, non-Hispanic Black, Hispanic, and non-Hispanic other. Income was a respondent’s estimate of total pre-tax income for the last year, including a spouse or partner if applicable. Missing income values were imputed by NHATS and included within the public use file (see Montaquila et al. [17] for details regarding imputation methodology). Income was categorized into six levels: less than $10,000, $10,001–$20,000, $20,001–$35,000, $35,001–$65,000, $65,001–$100,000, and >$100,000. Education was condensed into four categories: less than high school, high school diploma/general education development (GED) examination, some college, and a bachelor’s degree or greater. Similar to other published studies with NHATS data, we grouped respondents who did not identify their highest level of education with those who had less than high school [18, 19].

### Medical conditions

Respondents were asked whether a doctor had ever told them they had one of the following conditions: heart attack, heart disease, high blood pressure, arthritis, osteoporosis, diabetes, lung disease, stroke, Alzheimer’s disease or dementia, cancer (excluding skin), or a broken or fractured hip. The Patient Health Questionnaire-4 was administered to identify symptoms of depression and anxiety. Depression and anxiety were each scored on a scale of 0–6; a score of 3 or greater indicated the presence of symptoms for each condition [20]. Using these answers, we created a count of chronic conditions ranging from 0 to 13.

#### Functional status

In Round 1, NHATS included questions from the 2004 National Long-Term Care Survey (NLTCS) Screener on Activities of Daily Living (ADL) and Instrumental Activities of Daily Living (IADL) [21]. Functional limitation prevalence was estimated from this NLTCS module. Respondents who identified having a problem performing one of the following ADLs without help were considered to have a disability in that area: eating, getting in or out of bed, getting in or out of chairs, walking around inside, dressing, bathing, and toileting. If a respondent was not able to perform one of the following IADLs without help, they were considered to have a disability in that area: preparing meals, doing laundry, light housework, shopping for groceries, managing money, taking medicine, and making phone calls. If a person did not do an IADL activity but said they were able, the respondent was marked as having no disability in that area. A total count of ADL and IADL difficulties was calculated by adding up the number of limitations, creating a scale of 0–7 for both areas. There was also a separate binary measure of the ability to go outside without the help of another person or special equipment. We separated this from the total ADL count and analyzed it as an individual variable.

### Pain

Respondents were categorized as having pain if they answered yes to the question, “In the last month, have you been bothered by pain?” [22].

#### Outcomes

Level of participation, degree of importance, and barriers to participation:

Respondents were asked whether they had performed four activities in the last month: (1) visit friends and family who lived separately, (2) attend religious services, (3) participate in clubs, classes, or other organized activities, or (4) go out for enjoyment. They were also asked whether they experienced limitations performing the activities in the last month due to health or transportation issues, regardless of whether the activity was performed or not. These answers to these questions about limitations to the activities were the barriers. The answers were coded as yes or no. Respondents were also asked to rate the level of importance of performing these four activities: very important, somewhat important, or not so important. For our analyses, activities endorsed as somewhat or very important were combined and considered “valued activities” to the respondent. If respondents rated one of the four activities important, we examined whether they participated in the activity within the last month and what barriers they experienced to that participation. Respondents were also asked whether they provided care to another adult or child who could not care for themselves in the last month as this can be an important social outlet.

### Statistical analyses

We examined the demographic and socioeconomic characteristics, health conditions, and functional limitations of sample adults by homebound status. Each group was compared to the completely homebound group via Chi-square analysis for categorical variables and Student’s *t* test for continuous variables. To test the hypothesis that the majority of homebound older adults would report that community participation was important but that both intrinsic (individual) and extrinsic (environmental) factors present barriers to participation among older adults, we calculated frequencies of activity importance, participation, and barriers according to homebound status. Analyses included survey weights to adjust for the NHATS survey design and to generalize the national sample. All analyses were performed using Stata 13.1 (Stata Corp, College Station, TX).

## Results

### Study participants

Based on the Ornstein et al. measure of homebound status, our analytic sample was comprised of 473 homebound individuals, 1257 semi-homebound individuals, and 5467 non-homebound individuals. Applying population weights and extrapolating to the entire USA, the group who never or rarely left the house in the last month, defining our homebound sample, represents 1,551,121 older adults. The sample of those who found it difficult to leave the house represents 3,832,428, and the sample that did not leave without another person represents 960,255, totaling 4,792,683 semi-homebound individuals. Non-homebound individuals represent 27,011,310 older adults. The homebound sample mean age was 80.1 (SD = 9.80), and respondents were significantly more likely to report limitations in ADLs as well as IADLs than those who were not homebound (Table 1). The IADLs with which the highest percentage of homebound reported restrictions were shopping for groceries, doing laundry, and preparing meals, which have important implications for daily life and for community participation. Percentages for other IADL difficulties were managing money, light housework, taking medicine, and making phone calls. The top three ADL impairments for homebound individuals were bathing, walking around inside, and getting in or out of chairs, followed by dressing, getting in or out of bed, toileting, and eating.

**Table 1:** Respondent characteristics

Characteristic	Homebound (*n* = 473)	Semi-homebound (*n* = 1257)	Non-homebound (*n* = 5467)	Total (*n* = 7197)
Weighted total (*N*)	1,551,121	4,792,683	27,011,310	33,355,114
*Demographics*				
Age, mean (SD)	80.1 (9.80)	77.7 (8.50)**	74.0 (6.18)**	74.8 (6.89)
Gender (%)				
Male	25.8	34.7*	47.0**	44.3
Female	74.2	65.3	53.0	55.7
Education (%)				
<High school	48.1	36.7**	18.9**	22.8
High school/GED	26.3	25.9	27.3	27.1
Some college	17.9	23.6	26.8	25.9
≥Bachelor’s	7.7	13.8	27.0	24.2
Race (%)				
White, non-Hispanic	62.7	73.1**	82.3**	80.1
Black, non-Hispanic	13.6	11.1	7.5	8.3
Hispanic	17.8	10.9	5.6	6.9
Other	5.9	4.9	4.6	4.7
Income (%)				
≤$10,000	26.1	17.3**	8.4**	10.5
$10,001–$20,000	37.6	32.8	17.8	20.9
$20,001–$35,000	21.6	21.8	21.9	21.8
$35,001–$65,000	9.7	17.6	26.0	24.1
$65,001–$100,000	2.8	6.3	14.9	13.1
>$100,000	2.2	4.2	11.0	9.6
*Clinical and functional*				
Pain in the last month (%)	75.2	76.3	47.1**	52.6
Depression^a^ (%)	43.5	28.2**	9.8**	14.0
Chronic conditions, mean (SD)	4.7 (2.42)	4.0 (2.21)**	2.4 (1.52)**	2.8 (1.79)
Difficulty going outside (%)	60.9	42.6**	2.8**	11.2
Falls in the last month (%)	22.5	23.1	7.0**	10.0
Functional limitations, mean (SD)				
ADL impairments	2.3 (3.04)	1.2 (2.10)**	0.1 (0.48)**	0.3 (1.17)
Eating	17.3	6.2**	0.5**	2.1
Getting in or out of bed	31.4	16.6**	0.9**	4.6
Getting in or out of chairs	38.0	19.9**	1.2**	5.6
Walking around inside	41.0	23.9**	1.2**	6.3
Dressing	34.2	20.1**	1.2**	5.5
Bathing	42.5	22.6**	1.2**	6.2
Toileting	29.5	12.3**	0.7**	3.7
IADL impairments	3.6 (3.10)	2.0 (2.55)**	0.3 (1.12)**	0.7 (1.70)
Preparing meals	52.3	26.9**	4.2**	9.7
Doing laundry	60.1	35.2**	5.0**	11.9
Light housework	49.5	23.9**	3.4**	8.5
Shopping for groceries	73.7	49.1**	6.5**	15.7
Managing money	51.0	28.8**	6.0**	11.3
Taking medicine	40.6	24.2**	3.8**	8.5
Making phone calls	30.5	15.4**	3.0**	6.1

**p < 0.001; *p < 0.05. Homebound are reference group

^a^Depressive symptoms based on a PHQ-4 score ≥ 3

^b^Nominal data were missing with a range of 0–1.96 %

As described previously [9], non-homebound individuals were younger (74.0 [SD = 6.18] vs. 80.1 [SD = 9.80]), more likely to be male (47.0 vs. 25.8 %) and White (82.3 vs. 62.7 %) than those who are homebound. They are also significantly more educated and have higher income than those who are completely homebound. Non-homebound individuals are much less likely to have depressive symptoms (9.8 vs. 43.5 %) than the homebound and less likely to report having been bothered by pain in the last month (47.1 vs. 75.2 %).

### Community participation

**Fig. 1:**
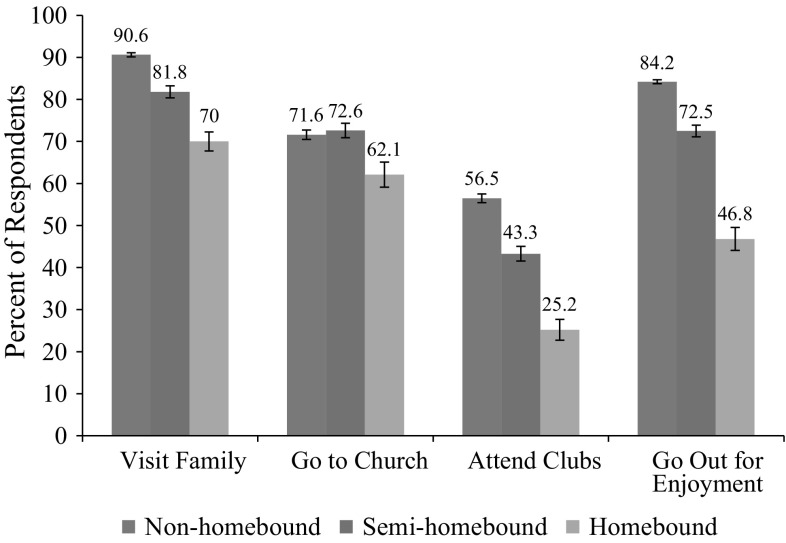
Activity importance by group

Despite these functional limitations and pain, homebound and semi-homebound older adults report a strong interest in community participation (see Fig. 1). The homebound (those who never or rarely left the house in the past month) frequently report that activities outside the home were important to them ranging from 25.2 % (attend clubs) to 70.0 % (visit family). Similarly, a majority of the semi-homebound reports that visiting in person with friends and family (81.8 %), attending religious services (72.6 %), and going out for enjoyment (72.5 %) are important to them. A full 43.3 % report that participating in clubs, classes, and other activities is important to them.

### Barriers to participation

Despite this importance, homebound and semi-homebound individuals reported that health concerns often kept them from participating in various activities. Of those who endorsed the activities as important, many homebound reported health (42.9–64.1 % depending on the activity) and some reported transportation issues (12.2–18.2 %) as barriers to participation (see Table 2). Among the semi-homebound who reported that these activities were important to them, 23.8–41.0 % reported barriers due to health concerns and 6.5–10.2 % reported transportation barriers. Interestingly, 6.6 % homebound older adults provide care for others, and 9.7 % of the semi-homebound provide care for others.

**Table 2:** Performance in the last month and barriers to activities people valued as important

	Not done in past month (%) [SE]	Health prevented activity (%) [SE]	Transportation prevented activity (%) [SE]
*Homebound*			
Visit in person with friends or family	21.7 (2.73)	42.9 (2.97)	16.1 (2.45)
Attend religious services	59.7 (3.05)	64.1 (3.05)	14.0 (2.47)
Participate in clubs, classes, or other activities	70.5 (4.79)	58.4 (5.52)	18.2 (4.69)
Go out for enjoyment	54.3 (3.76)	59.3 (4.17)	12.2 (2.57)
*Semi*-*homebound*			
Visit in person with friends or family	15.8 (1.57)	23.8 (1.51)	8.3 (0.97)
Attend religious services	36.7 (1.50)	41.0 (1.87)	10.2 (1.00)
Participate in clubs, classes, or other activities	54.0 (2.70)	34.1 (2.58)	8.4 (1.27)
Go out for enjoyment	19.3 (1.68)	26.0 (1.58)	6.5 (1.08)
*Non*-*homebound*			
Visit in person with friends or family	6.5 (0.47)	3.5 (0.22)	1.3 (0.16)
Attend religious services	20.0 (0.76)	7.4 (0.45)	1.8 (0.20)
Participate in clubs, classes, or other activities	30.9 (1.09)	5.7 (0.54)	1.0 (0.14)
Go out for enjoyment	8.4 (0.53)	4.0 (0.28)	0.9 (0.12)

## Discussion

### Findings

To our knowledge, this is the first study to report on valued family and community-based activities by community-dwelling homebound older adults using nationally representative data. We found that despite functional limitations, large percentages of homebound and semi-homebound older adults seek to participate in family and community life. Community participation can be an important part of health promotion [23], can decrease depression [24], and is actionable, which makes it an important intervention target.

As we hypothesized, we found that homebound older adults have more activity-limiting health problems (intrinsic factors) than their non-homebound counterparts. They also have transportation issues (extrinsic factors). These patterns are relevant because it is both harder to reach homebound older adults and more important to understand what deficits they need to overcome to engage in the community. *It is important to note that in our sample, the homebound had the lowest percentage of valued activities of the three groups.* On the positive side, we found that both the homebound and the semi-homebound are able to see family frequently. That was the most highly valued activity and is accomplished by 78.3 % of the homebound and 84.2 % of the semi-homebound. The semi-homebound group was the larger of the groups. This is a less restrictive definition and combines two subgroups of individuals that may not necessarily progress to homeboundedness—those who need help to go out and those who do not go out unaccompanied. The semi-homebound is potentially the more robust group for intervention because they are healthier, have less impairment, and have more available social support. Finally, from our findings, shopping for groceries, doing laundry, and preparing meals were the most common self-care difficulties. Each one of these is more amenable to outside help than ADLs like bathing and grooming. Targeted programs such as meals on wheels and also light housecleaning and laundry could provide semi-homebound or homebound older adults the ability to participate in valued activities.

### Findings in context

Our findings add to the literature on homebound older adults’ participation preferences. Previous studies have examined single communities. Lack of opportunity for social and community engagement is among their prominent findings. Murayama et al. [25] found that walkability and crime safety affect community participation of homebound older adults in a Japanese community. Turcotte et al. [12] found that older disabled adults were not receiving enough social participation opportunities. Both home and community adaptations are associated with community participation among mobility-limited older adults in the US State of Georgia [26]. Bendixen et al. [11] found that those who cannot participate suffer lower self-esteem and may subsequently have more role losses. Hammel et al. [27] found that toilet and bath modifications (even more than ramps or lifts for entrance/egress) had the largest association with going out into the community for those with mobility limitations. Our study findings extend those of others in their nationally representative scope.

### Limitations

The current study has important limitations. First, although the definition of homebound and semi-homebound has strong convergent validity with illness and the same definitions have been used by others [9, 25], the measure identifying homebound older adults is based on whether respondents left the house in the last month. This question has seasonality issues as someone in a cold wintry or hot summer locale may not leave in the recent month but not be considered homebound during temperate weather. Or, theoretically, it could have been an unusual month in an otherwise active life. This seems unlikely because an unusually weakened older adult would not likely volunteer for a 3-h research interview, but it is possible. A second limitation to our findings is the possible endogeneity. People may be more likely to say that an activity is important to them if they were able to have done it recently. Similarly, transportation difficulty could be confounded with homebound status because respondents could have answered that they did not go out due to transportation. *Also, we are unable to determine whether the fact that homebound older adults value participation less than the non*-*homebound is due to other factors beyond the existence of intrinsic and extrinsic barriers to participation (e.g., overall interest).* While the sample size of 473 is modest, this is a nationally representative sample of 473 people representing 1.5 million adults. Further, this sample size is larger than the overwhelming majority of previous work on homebound older adults who are especially difficult to recruit to research studies because of their poor health and inability to access routine medical care or other services. Also, those who were homebound were less educated than those who were not homebound. There may be a cohort effect as new generations of older adults are more educated.

### Participation in general

For an aging society, having 6.3 million homebound and semi-homebound older adults who want to participate in societal life can be an opportunity. In recent years, many communal networks such as the village model and naturally occurring retirement communities have sprung up to meet community needs. If homebound and semi-homebound adults can more easily leave the house, they may be able to contribute to these communities. There are also new Internet-based options such as Skype, Magic Window, and Virtual Senior Centers which can remove barriers to participation using electronic connections.

For those who are semi- or fully homebound and want to get out into the community, their vulnerability makes their safe participation more difficult to facilitate. The high risk of falls in the homebound is of particular import as we think societally of how best to facilitate their social and community engagement. It will be important to target environmental needs [28] and other services that might facilitate leaving home to participate in community events such as mobility services and other para-transit services.
